# Endogenous assessment of myocardial injury with single-shot model-based non-rigid motion-corrected T1 rho mapping

**DOI:** 10.1186/s12968-021-00781-w

**Published:** 2021-10-21

**Authors:** Aurélien Bustin, Solenn Toupin, Soumaya Sridi, Jérôme Yerly, Olivier Bernus, Louis Labrousse, Bruno Quesson, Julien Rogier, Michel Haïssaguerre, Ruud van Heeswijk, Pierre Jaïs, Hubert Cochet, Matthias Stuber

**Affiliations:** 1 INSERM, Centre de Recherche Cardio-Thoracique de Bordeaux, U1045, IHU LIRYC, Electrophysiology and Heart Modeling Institute, Université de Bordeaux, Avenue du Haut Lévêque, 33604 Pessac, France; 2 Department of Cardiovascular Imaging, Hôpital Cardiologique du Haut-Lévêque, CHU de Bordeaux, Avenue de Magellan, 33604 Pessac, France; 3 Department of Diagnostic and Interventional Radiology, Lausanne University Hospital and University of Lausanne, Lausanne, Switzerland; 4 Siemens Healthcare France, 93210 Saint-Denis, France; 5 Center for Biomedical Imaging (CIBM), Lausanne, Switzerland; 6 Department of Cardiac Surgery, Hôpital Cardiologique du Haut-Lévêque, CHU de Bordeaux, Avenue de Magellan, 33604 Pessac, France; 7 Department of Cardiac Electrophysiology, Hôpital Cardiologique du Haut-Lévêque, CHU de Bordeaux,, Avenue de Magellan, 33604 Pessac, France

**Keywords:** Parameter mapping, Myocardial, T1ρ mapping, Model-based, Non-rigid, Motion correction

## Abstract

**Background:**

Cardiovascular magnetic resonance T1ρ mapping may detect myocardial injuries without exogenous contrast agent. However, multiple co-registered acquisitions are required, and the lack of robust motion correction limits its clinical translation. We introduce a single breath-hold myocardial T1ρ mapping method that includes model-based non-rigid motion correction.

**Methods:**

A single-shot electrocardiogram (ECG)-triggered balanced steady state free precession (bSSFP) 2D adiabatic T1ρ mapping sequence that collects five T1ρ-weighted (T1ρw) images with different spin lock times within a single breath-hold is proposed. To address the problem of residual respiratory motion, a unified optimization framework consisting of a joint T1ρ fitting and model-based non-rigid motion correction algorithm, insensitive to contrast change, was implemented inline for fast (~ 30 s) and direct visualization of T1ρ maps. The proposed reconstruction was optimized on an ex vivo human heart placed on a motion-controlled platform. The technique was then tested in 8 healthy subjects and validated in 30 patients with suspected myocardial injury on a 1.5T CMR scanner. The Dice similarity coefficient (DSC) and maximum perpendicular distance (MPD) were used to quantify motion and evaluate motion correction. The quality of T1ρ maps was scored. In patients, T1ρ mapping was compared to cine imaging, T2 mapping and conventional post-contrast 2D late gadolinium enhancement (LGE). T1ρ values were assessed in remote and injured areas, using LGE as reference.

**Results:**

Despite breath holds, respiratory motion throughout T1ρw images was much larger in patients than in healthy subjects (5.1 ± 2.7 mm vs. 0.5 ± 0.4 mm, P < 0.01). In patients, the model-based non-rigid motion correction improved the alignment of T1ρw images, with higher DSC (87.7 ± 5.3% vs. 82.2 ± 7.5%, P < 0.01), and lower MPD (3.5 ± 1.9 mm vs. 5.1 ± 2.7 mm, P < 0.01). This resulted in significantly improved quality of the T1ρ maps (3.6 ± 0.6 vs. 2.1 ± 0.9, P < 0.01). Using this approach, T1ρ mapping could be used to identify LGE in patients with 93% sensitivity and 89% specificity. T1ρ values in injured (LGE positive) areas were significantly higher than in the remote myocardium (68.4 ± 7.9 ms vs. 48.8 ± 6.5 ms, P < 0.01).

**Conclusions:**

The proposed motion-corrected T1ρ mapping framework enables a quantitative characterization of myocardial injuries with relatively low sensitivity to respiratory motion. This technique may be a robust and contrast-free adjunct to LGE for gaining new insight into myocardial structural disorders.

**Supplementary Information:**

The online version contains supplementary material available at 10.1186/s12968-021-00781-w.

## Background

Research on quantitative magnetic resonance imaging has led to a greater understanding of the biochemical properties of human tissues. In particular, T1 rho (T1ρ) mapping has revealed new insights about the macromolecular content of biological tissues by showing substantial sensitivity to static processes and proton exchange and has added new information about compositional changes in human spinal disc degeneration, knee osteoarthritis, brain disease and liver fibrosis [1–3]. The idea that T1ρ mapping can be used to quantify myocardial fibrosis without the injection of contrast agent has offered the potential to transform the way we perform cardiovascular magnetic resonance (CMR).

The T1ρ relaxation describes the spin–lattice relaxation time in the rotating frame and was first introduced in vivo by Martino and Damadian in 1984 [4]. T1ρ measures the transverse relaxation in the presence of a continuous-wave radiofrequency (RF) pulse, also known as a spin lock (SL) pulse. The T1ρ weighting of the image is controlled by the duration (TSL) and frequency (FSL) of the SL pulse. T1ρ maps can be generated by fitting a series of T1ρ-weighted images, acquired with different TSL times, to a mono-exponential relaxation model.

Application of T1ρ mapping to CMR has been reported in multiple studies [5–8], mostly to discriminate between infarct and healthy myocardium in swine, mouse and monkey models. These studies raise the interesting question of whether endogenous T1ρ mapping could be a useful adjunct to late gadolinium enhancement (LGE) imaging. However, the evidence for a T1ρ elevation in the injured myocardium is largely based on controlled animal models and it remains unclear how it performs in patients due to the lack of clinical results. The sparing use of myocardial T1ρ mapping in clinical routine can at least partly be attributed to remaining technical challenges. An important limitation is the compensation for the complex motion of the heart associated with patient respiration. If no motion correction strategies are employed, severe breathing artefacts can considerably impact the quality of the reconstructed maps [9]. Breath-holding can be used to compensate for respiratory motion in myocardial T1ρ mapping [10, 11]. However, residual diaphragmatic drift is frequently observed, particularly in cardiac protocols that require multiple repetitive breath-holds [12]. Robust motion correction strategies are thus needed. Unfortunately, the unique capability of parameter mapping techniques, and particularly T1ρ mapping, to produce images with different contrast weightings, also represents a major challenge for intensity-based non-rigid registration algorithms. Indeed, such algorithms usually fail with multi-contrast images, where the brightness constancy assumption is not valid anymore.

Several advanced motion-correction techniques have been proposed to alleviate the influence of motion in other myocardial mapping applications such as T1 and T2 mapping. Techniques based on local non-rigid registration approaches, where a variational framework is employed to simultaneously estimates intensity changes and motion fields have been successfully employed in vivo [13, 14]. Groupwise motion correction techniques take a step further by eliminating the need of selecting a reference image for registration by describing the parameter dimension as a low-dimensional space through principal component analysis [15]. This technique was later successfully applied to T1 mapping in patients [16].

While these techniques have been validated for several cardiac mapping applications, none of these approaches have been applied to myocardial T1ρ mapping and advancing the clinical translation of myocardial T1ρ mapping thus requires the engineering of specialized motion correction strategies.

This study investigates a novel inline model-based non-rigid motion correction technique for myocardial T1ρ mapping that makes use of the known signal model to drive the motion correction process. The performance of the developed framework is first optimized in ex vivo human heart using a motion-controlled experimental setup. The efficiency of the proposed motion corrected myocardial T1ρ mapping is then assessed in 8 healthy subjects without history of cardiovascular disease. Finally, its clinical feasibility is investigated prospectively in a cohort of 30 patients with a broad range of ischemic and non-ischemic cardiomyopathies.

## Methods

Ex vivo and in vivo acquisitions were performed on two 1.5T CMR scanners (MAGNETOM Aera, software version syngo.MR VE11C, for ex vivo human heart and patients and MAGNETOM Sola, software version syngo.MR XA20, for healthy subjects, Siemens Healthineers, Erlangen, Germany) with a dedicated 32-channel spine coil and an 18-channel phased-array coil. The study was approved by our institutional review board and all healthy subjects and patients gave informed consent. Image analysis was performed offline using MATLAB (v9.7, The MathWorks, Natick Massachusetts, USA).

### Myocardial T1ρ mapping acquisition

**Fig. 1:**
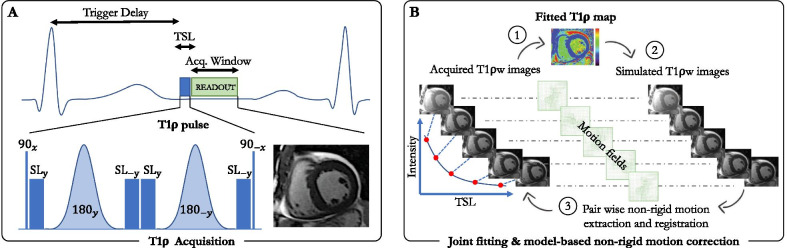
Schematic of the proposed single breath-hold 2D myocardial T1ρ mapping technique (**A**) with joint T1ρ fitting and model-based motion correction (**B**). T1ρ mapping is performed using a single-shot electrocardiogram (ECG)-triggered balanced steady-state free precession acquisition where five images with different spin lock times (TSL) are acquired over 13 heartbeats within a single breath-hold. The T1ρ preparation pulse includes 4 spin locks and 2 adiabatic refocusing pulses. Motion correction is performed by iterating between a Levenberg–Marquardt T1ρ fitting, generating a T1ρ map used for the simulation of T1ρ-weighted images. These simulated images have the same contrast than the acquired images and a pair-wise non-rigid motion estimation can thus be performed between the two sets of images. The obtained non-rigid motion fields are then used to transform the acquired images to the same motion state, and these steps are repeated until convergence

The acquisition method is illustrated in Fig. 1A. An electrocardiogram (ECG)-triggered 2D single-shot (i.e., one image per heartbeat), balanced steady-state free-precession (bSSFP) T1ρ mapping sequence was implemented on our 1.5 T CMR systems. The sequence incorporates an adiabatic T1ρ preparation module to achieve T1ρ-weighted images. This T1ρ module first plays out a $90^\circ$ tip-down pulse along the x-axis to rotate the magnetization, followed by four spin-lock pulses with alternating phases (${\mathrm{SL}}_{\pm y}$) and fixed duration, and two adiabatic refocusing pulses (${180}_{\pm y}$). An additional $90^\circ$ tip-up pulse is played out to return the magnetization to the z-axis resulting in the cluster (${90}_{x}-{\mathrm{SL}}_{\mathrm{y}}-{180}_{y}-{\mathrm{SL}}_{-y}-{\mathrm{SL}}_{y}-{180}_{-y}-{\mathrm{ SL}}_{-y}-{90}_{-x}$). Adiabatic pulses were employed for their reduced sensitivity to a broad range of B0 and B1 field inhomogeneities [17, 18] (see Additional file 1). The rotation angle of the spin-lock components was defined by ${\mathrm{\alpha }}_{\mathrm{SL}}=2\pi \times FSL\times TSL$, where FSL is the spin-lock frequency and TSL is the total duration of the spin-lock pulses. A crusher gradient is then used to remove any residual transverse magnetization. The amplitude of the spin-lock RF pulse was set to 500 Hz, following the literature [5, 11]. Five T1ρ-weighted images (${N}_{TSL}=5$) with different TSL [(0, 10, 20, 35, 50) ms] were acquired sequentially in mid-diastole during 13 heartbeats (repetition time of 3 heartbeats to allow for full magnetization recovery) in a single breath-hold. The trigger delay was adapted for each of the five images to ensure that the image readout was always in the same cardiac phase.

### Joint T1ρ fitting and model-based non-rigid motion correction

The motion correction method is illustrated in Fig. 1B. In the ideal case, a fitting model can be employed to generate the myocardial T1ρ map. However, non-rigid respiratory drift may occur during the single-shot T1ρ-weighted image acquisition and will adversely affect the final T1ρ map. Therefore, an advanced motion correction strategy that can handle multi-contrast imaging needs to be developed. Here, we address the problem of such residual respiratory motion using a joint T1ρ fitting and model-based non-rigid motion correction algorithm, insensitive to contrast change, to efficiently reconstruct motion-corrected 2D myocardial T1ρ maps. The proposed respiratory motion correction framework is shown in Fig. 1B and can be formulated as the following optimization problem:

1 $$\mathop {\min }\limits_{\theta ,p} \mathop \sum \limits_{t = 1}^{{N_{TSL} }} || f_{t} \left( p \right) - E_{{\theta_{t} }} y||_{2}^{2} + \mu ||Gp||_{2}^{2} + \lambda S\left( {\theta_{t} } \right)$$

where $y$ are the acquired multi-contrast single-shot images, $$p=\left(\begin{array}{c}\mathrm{T}1\uprho \\ {M}_{0}\end{array}\right)$$ are the unknown parameters to recover, with ${M}_{0}$ depicting the initial longitudinal magnetization, T1ρ the final map and ${f}_{t}\left(p\right)={M}_{0}\mathrm{exp}\left(-\frac{TS{L}_{t}}{\mathrm{T}1\uprho }\right)$ our two-parameter fitting model. The warping operator $E$ describes a non-rigid deformation ${\theta }_{t}$ for each image $t$. Because non-rigid motion field estimation is an ill-posed inverse problem, a regularization term that penalizes the L2-norm of motion-field gradients was employed ($S\left({\theta }_{t}\right)={\Vert \nabla {\uptheta }_{t}\Vert }_{2}^{2}$). Furthermore, an additional spatial smoothness constraint $G$, returning the spatial gradients of each parameter map, was added to the parameter map to reduce local variations and make the technique more robust to noise. The two scalars $\mu$ and $\lambda$ denote the regularization weights. Having established the notations, Eq. [1] can then be solved by splitting the optimization into two easier sub-problems for $\theta$ and $\rho$:

2 $$\theta^{i + 1} = \mathop {{\text{argmin}}}\limits_{\theta } \mathop \sum \limits_{t = 1}^{{N_{TSL} }}|| f_{t} \left( {p^{i} } \right) - E_{{\theta_{t} }} y||_{2}^{2} + \lambda S\left( {\theta_{t} } \right)$$

3 $$p^{i + 1} = \mathop {{\text{argmin}}}\limits_{p} \mathop \sum \limits_{t = 1}^{{N_{TSL} }}|| f_{t} \left( p \right) - E_{{\theta_{t}^{i + 1} }} y||_{2}^{2} + \mu Gp_{2}^{2}$$

The sub-problem in Equation [2] now consists of a typical pair-wise non-rigid motion field estimation between the synthetic images ${f}_{t}\left({p}^{i}\right)$ and the acquired images $y$ with $p$ being fixed, thus making the registration problem insensitive to contrast change (see Fig. 1B—step 3). This optimization can be solved in a multi-resolution manner, so that the displacement fields are first calculated at a coarser scale, then interpolated to the next (finer) resolution level [19]. The process is repeated until convergence at the finest scale, as reported in Odille et al. [20, 21]. With $\theta$ being fixed, Equation [3] becomes a data fitting problem that can be solved efficiently using a vectorized Levenberg–Marquardt algorithm as documented in Liu et al. [22]. Repeating these two steps iteratively provides the final solution of Eq. [1] after reaching a certain number of iterations.

### Implementation

To assess the clinical performance of the proposed myocardial T1ρ mapping framework, the model-based non-rigid motion-correction algorithm was written in C + + and integrated into the scanner reconstruction software (Image Calculation Environment, Siemens Healthineers). The reconstruction starts directly after the acquisition of the T1ρ-weighted images and sends four sets of images (corrected and non-corrected T1ρ-weighted images and respective T1ρ maps) to the local PACS workstation. Reconstruction parameters for the proposed approach were empirically optimized in the ex vivo study and were maintained for all in vivo reconstructions. Regularization parameters $\mu$ and $\lambda$ were empirically set to 0.01 and 0.008, respectively. Five outer iterations were performed and a number of four pyramid levels were set for the registration. The average time for the full reconstruction was ~ 30 s.

### Ex vivo study

**Fig. 2:**
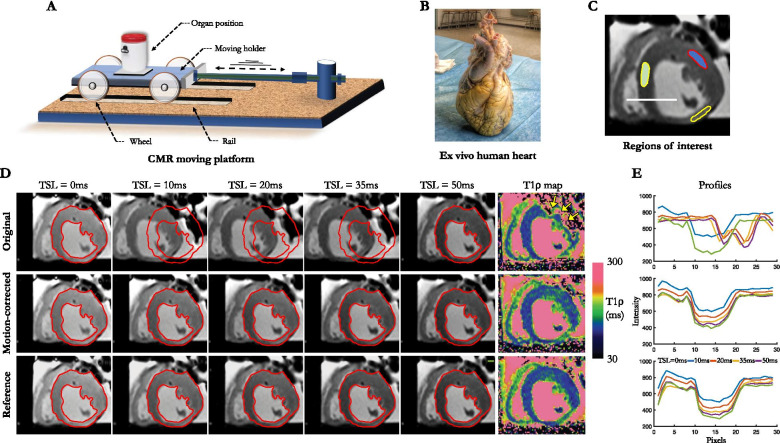
**A** Ex vivo moving platform consisting of a moving holder with four wheels moving on rails through an actuator inducing breathing motion. The ex vivo human heart **B** is placed on the trolley holder. **C** For T1ρ value assessment, regions of interest are manually drawn on the septal and anterolateral segments of the left ventricle. **D** Original, motion-corrected and motion-free reference T1ρ-weighted images and their corresponding T1ρ maps. **E** Signal intensity profiles are extracted from the T1ρ-weighted images

The ex vivo experiment is illustrated in Fig. 2. To understand how the proposed motion-corrected T1ρ sequence performs in a motion-controlled environment, we designed a CMR-compatible setup consisting of a moving Plexiglas trolley positioned on four wheels and connected to an actuator to induce breathing motion (Fig. 2A). A human heart with prior myocardial infarction was obtained from a donor with informed consent from family members, with approval from the National Biomedical Agency and in a manner conforming to the Declaration of Helsinki. The organ was procured at the Bordeaux University Hospital, transported in ice cold cardioplegia to the laboratory and fixed in formalin (Fig. 2B). The ex vivo heart was placed on the trolley holder to simulate a breathing movement. The trolley was consistently moved in a superior-inferior direction relative to the heart at a frequency of 15 cycles/min with a maximum amplitude of 18 mm as previously observed in vivo [23]. Static ground-truth T1ρ maps were acquired for comparison purposes prior to inducing motion. Scan parameters for myocardial T1ρ mapping were: in-plane resolution = 1.4 × 1.4 mm^2^, field-of-view = 190 × 220 mm^2^, slice thickness = 8 mm, one mid-ventricular short-axis slice, flip angle = 70°, TE/TR = 1.2/2.7 ms, phase partial Fourier = 6/8, 72 segments, acceleration GRAPPA 2 with 36 reference k-space lines, bandwidth = 1149 Hz/px, three recovery heartbeats, 13 heartbeats per slice, TSL = [0, 10, 20, 35, 50] ms and FSL = 500 Hz. Imaging was performed at a simulated heart rate of 60 beats per minute.

### Healthy subject study

To understand how the sequence performs in vivo and whether the motion correction impacts the T1ρ values, eight healthy subjects (three females; mean 30 years; range 24–40 years) without a history of cardiac disease were scanned with the proposed sequence. The same parameters as in the previous ex vivo study were used except that 3 short axis slices were acquired (basal, mid, and apical). The T1ρ mapping sequence was acquired during breath holds and timed such that the data acquisition occurred at mid-diastole.

### Patient study

A patient study was performed to assess the performance of the sequence in a more challenging population and to validate its integration in a clinical workflow. From July 2020 to October 2020, 30 adult patients (six females; mean 59 years; range 18–80 years) referred for CMR at Haut-Lévêque Hospital in Bordeaux, France were prospectively included. Patients were not consecutive as the inclusion depended on the clinical workflow and was also impacted by competing research projects on similar patients. The inclusion criterion was an indication for CMR as part of standard care. Exclusion criteria were any contraindications to CMR. The CMR protocol consisted of cine bSSFP imaging (in contiguous short axis slices covering the whole ventricles, as well as in 2-, 3- and 4-chamber orientations), pre-contrast T1$\uprho$ and T2 mapping (both acquired in 3 short axis slices at basal, mid, and apical levels). A 4-chamber T1$\uprho$ map was also acquired in one patient (Fig. 7c—patient 3). Post-contrast 2D LGE imaging was performed 12 min after injection of 0.2 mmol/kg gadoteric acid in 3 series of contiguous slices covering the whole left ventricle (LV) in short axis, 2-chamber and 4-chamber orientations. Imaging parameters for the mapping and LGE sequences are summarized in Table 1. All images were acquired during breath holds. For pre-contrast myocardial T1ρ mapping, imaging parameters were identical to those used in volunteers.

**Table 1:** Descriptions of the CMR sequences employed in the patient study

Sequence	T2 MAPPING	T1ρ MAPPING	LGE
Acquisition	Non-selective T2-prepared bSSFP	Non-selective T1ρ-prepared bSSFP	Non-selective IR GRE
Magnetic field, Tesla	1.5	1.5	1.5
Post-contrast	No	No	Yes
Acceleration	GRAPPA 2	GRAPPA 2	GRAPPA 2
Reconstructed resolution, mm^2^	1.9 × 1.9	1.4 × 1.4	1.5 × 1.5
Slice thickness, mm	8	8	4
Number of LV slices, range	3	3	11–16
ECG triggering (RR)	3	3	2
TR/TE, msec	2.5/1.1	2.7/1.2	3.9/1.7
Bandwidth, Hz/pixel	1184	1150	362
Flip angle, degrees	70	70	10
Free breathing	No	No	No

*bSSFP* balanced steady-state free precession, *ECG* electrocardiogram, *GRE* gradient echo; *LGE* late gadolinium enhancement, *IR* inversion recovery, *GRAPPA* generalized autocalibrating partially parallel acquisitions, *LV* left ventricle, *TR* repetition time, *TE* echo time

### Data analysis

To quantify motion and assess the performance of motion correction in the ex vivo heart, healthy subjects and patients, epicardial and endocardial myocardial boundaries were drawn on each T1ρ-weighted image (non-corrected and motion-corrected), using a custom MATLAB software. The Dice similarity coefficient (DSC) [24] was calculated as a measure of registration accuracy (spatial overlap) throughout the T1-weighted image series, and was averaged over all image pairs $\left(i,j\right)$:

4 $$DSC_{s} \left( {i,j} \right) = \frac{{2 \cdot \left( {A_{i} \cap A_{j} } \right)}}{{A_{i} + A_{j} }}$$

5 $$DSC = \frac{100}{{N_{TSL} \times \left( {N_{TSL} - 1} \right)}}\mathop \sum \limits_{i = 1}^{{N_{TSL} }} \mathop \sum \limits_{j = 1,j \ne i}^{{N_{TSL} }} DSC_{s} \left( {i,j} \right)$$

where ${A}_{i}$ is the myocardium segmentation of image $i$. In the case of perfect registration, the DSC would be equal to 100% whereas a DSC value near 0% would indicate poor registration. In addition, the maximum perpendicular distance (MPD, also called Hausdorff distance) between segmented contours was measured. This metric calculates the maximum displacement of endocardial and epicardial contours across the T1ρ-weighted images. MPD is computed in millimeter per subject and per image set (before and after motion-correction), with a high value indicating a broader displacement due to respiratory drift despite breath-hold. The reproducibility of DSC and MPD was assessed from 2 distinct segmentations performed on the entire participants population by two independent expert scientists (S.T. and A.B., 6 and 7 years of CMR experience, respectively) blinded to the registration results.

*In the *ex vivo* heart*, T1ρ values were extracted from 3 region of interests (ROIs) drawn on the septum and the remote LV free wall, as well as within the infarcted area exhibiting wall thinning. These were compared to ground truth values from the static acquisition. Signal intensity profiles along a transmural septal radius (white line in Fig. 2C) were extracted and visually compared to ground truth profiles to assess the degree of mismatch between T1ρ-weighted images. *In healthy subjects*, the influence of motion correction on myocardial T1ρ values was assessed by drawing circumferential transmural ROIs on mid-ventricular slices with and without motion correction. *In patients*, epicardial and endocardial contours were manually drawn on cine images at end-diastole and end-systole using a dedicated software (cvi42, Circle Cardiovascular Imaging, Calgary, Alberta, Canada) to assess LV volume and ejection fraction (LVEF). Two experienced radiologists (H.C. and S.S., 18 and 5 years experience in CMR, respectively) qualitatively analysed cine, T1ρ maps, T2 maps and LGE images in consensus. Assessment of segmental wall motion abnormality was visually performed, and segments were graded as 0 for normal; 1 for mild or moderate hypokinesia; 2 for severe hypokinesia; 3 for akinesia; and 4 for dyskinesia [25]. Injured areas were defined as regions with LGE (based on the 2 SD segmentation method [26]), while remote areas were defined as regions with no LGE. The agreement between cine, T1ρ map, T2 map and LGE was assessed at a segmental level. The sensitivity and specificity of T1ρ to detect myocardial injury was assessed on a patient basis. Patients whose LGE areas were not covered by T1ρ and T2 mapping were excluded from this analysis. Quantitative analysis was achieved by tracing a ROI on T1ρ maps within remote and injured areas. The size of the ROIs in remote areas was 90 mm^2^ (corresponding to about 65 pixels). In the injured areas, the size of the ROIs was dictated by the boundaries of the scar. Segments on T1ρ and T2 maps were considered to be positive if at least a 2 SD increase of relaxation times above the mean remote value exist. In addition, to assess the impact of motion correction on myocardial T1ρ visualization, T1ρ maps were reviewed blinded to the particular reconstruction strategy, and image quality was graded using a 4-point scale (1-nondiagnostic with severe motion artifacts, 2-less than adequate with large motion artifacts, 3-adequate with moderate motion artifacts, 4-excellent image quality with no motion artifact).

### Statistical analysis

Continuous variables are expressed as mean ± standard deviation (SD), and categorical variables as fraction (%). Continuous variables were compared using parametric or non-parametric tests, depending on data normality. Paired t-tests were used to compare measurements performed with vs. without motion correction. Inter-expert reproducibility in DSC and MPD measurements was assessed using the ICC, along with mean bias and 95% limits of agreement. Statistical analysis was performed using SPSS (version 26.0, Statistical Package for the Social Sciences, International Business Machines, Inc., Armonk, New York, USA). A P value < 0.05 was considered to indicate statistical significance.

## Results

### Ex vivo study

Figure 2D shows the original, motion-corrected and motion-free reference T1ρ-weighted images ex vivo and their corresponding T1ρ maps. Superior image quality is obtained after motion-correction, with signal intensity profiles closer to the ground truth profiles extracted from the static acquisition (Fig. 2E). DSC score increased after motion-correction (94.8% vs. 49.7%) and was closer to the ground truth (96.4%). MPD decreased after motion correction (0.7 mm vs. 10.2 mm) and was closer to the ground truth (0.7 mm). T1ρ values were similar between non-corrected, motion-corrected, and ground truth maps in the septum (non-corrected: 104 ± 7 ms, motion-corrected: 102 ± 6 ms, ground truth: 103 ± 6 ms), but differed substantially in the free wall (non-corrected: 212 ± 137 ms, motion-corrected: 114 ± 5 ms, ground truth: 110 ± 6 ms), as visually expected from the T1ρ maps in Fig. 2D. T1ρ values were higher in the infarcted vs. remote septal areas on both the ground truth map (119 ± 12 ms vs. 103 ± 6 ms) and the motion-corrected map (121 ± 8 ms vs. 102 ± 6 ms). On the non-corrected map, T1ρ values in the infarct were not assessable due to major motion artifacts in the area.

### Healthy subjects study

**Fig. 3:**
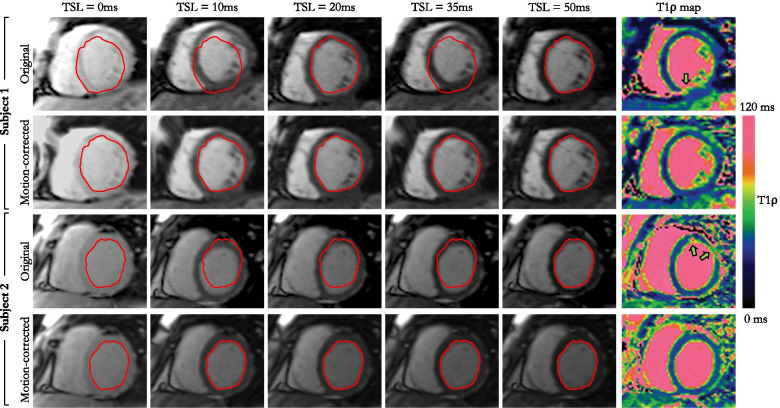
Comparison of myocardial T1ρ-weighted images and T1ρ maps before and after application of the proposed model-based non-rigid motion correction technique in two healthy subjects

Over breath-held acquisitions, the MPD across T1ρ-weighted images without motion correction was 0.5 ± 0.4 mm in healthy subjects. Figure 3 and Additional file 2 show the T1ρ-weighted images and corresponding maps from four healthy subjects who particularly failed holding their breath. Overall, in healthy subjects, the use of motion-correction slightly improved the quality of the T1ρ-weighted images resulting in sharper T1ρ maps. However, motion correction did not significantly improve DSC (81.1 ± 4.9% vs. 77.9 ± 15.5%, p = 0.58) or MPD (0.4 ± 0.2 mm vs. 0.5 ± 0.4 mm, P = 0.50). Likewise, there was no significant difference in T1ρ values (mean T1ρ with correction: 47.7 ± 4.0 ms, without correction: 48.9 ± 4.2 ms, P = 0.56) and precision (T1ρ SD with correction: 1.4 ± 0.4 ms, without correction: 1.6 ± 0.6 ms, P = 0.53).

### Patient population

Baseline characteristics of the patient cohort (N = 30) are shown in Table 2. The mean LVEF was 50 ± 14%. The final diagnosis was negative CMR in 6 (20%), ischemic heart disease in 10 (33%), and non-ischemic heart disease in 14 (47%), including 4 (13%) myocarditis, 3 (10%) hypertrophic cardiomyopathy, 4 (13%) dilated cardiomyopathy, 1 (3%) arrhythmogenic cardiomyopathy with LV involvement, 1 (3%) Takotsubo cardiomyopathy and 1 (3%) amyloidosis. Myocardial LGE was found in 22 patients (73%). T2 imaging showed acute edema in 4 (13%) patients.

**Table 2:** Baseline characteristics of the patient cohort (n = 30)

Patient characteristics	
Gender [F/M]	6/24
Mean age [years]	59 ± 16
Mean heart rate [beats/min]	59 ± 11
Mean body mass index [kg/m^2^]	24 ± 3
CMR diagnosis	
Myocardial infarction	10 (33%)
Dilated cardiomyopathy	4 (13%)
Myocarditis	4 (13%)
Hypertrophic cardiomyopathy	3 (10%)
Takotsubo cardiomyopathy	1 (3%)
Arrhythmogenic right ventricular cardiomyopathy	1 (3%)
Amyloidosis	1 (3%)
Negative CMR	6 (20%)
Cardiac function	
LVEF [%]	50 ± 14
Impaired LVEF	6 (20%)
LV EDV/BSA [ml/m^2^]	99 ± 19
LV ESV/BSA [ml/m^2^]	51 ± 20
Tissue characterisation	
Positive myocardial LGE	22 (73%)
Positive T2 mapping	4 (13%)
Positive T1ρ mapping	15 (50%)

Data are expressed as mean ± standard deviation unless otherwise specified. *LVEF* left ventricular ejection fraction, *EDV* end-diastolic volume, *BSA* body surface area, *ESV* end-systolic volume, *LGE* late gadolinium enhancement, *LVEF* left ventricular ejection fraction

### Motion correction of T1ρ images in patients

**Fig. 4:**
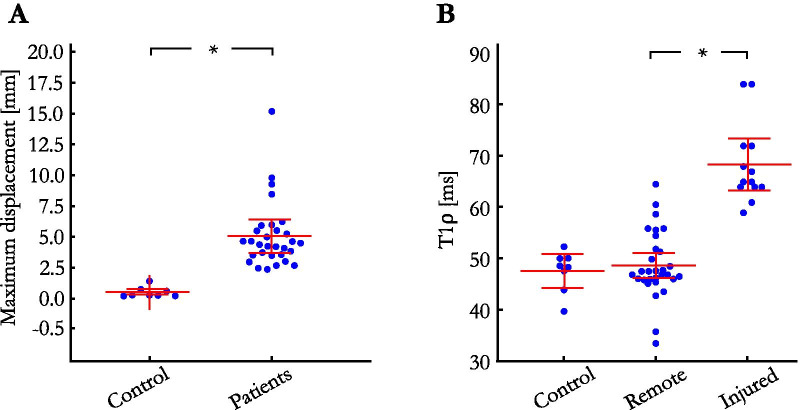
**A** Maximum displacement observed in the healthy subject and patient cohorts. **B** Remote and injured myocardial T1ρ values in the patient cohort. Horizontal lines depict means ± 95% confidence intervals. Statistical differences are indicated by *

**Fig. 5:**
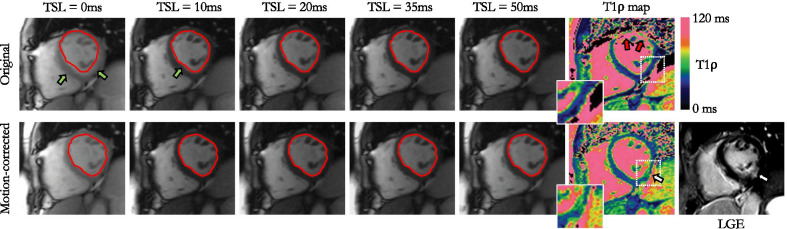
49-year-old female patient with inferolateral myocardial infarction visible on LGE and motion-corrected myocardial T1ρ mapping. The motion-corrected T1ρ map reveals a marked T1ρ elevation (64.9 ± 7.0 ms vs. 47.1 ± 3.8 ms) in the inferolateral segment of the left ventricle, in good agreement with the corresponding short-axis LGE image

**Fig. 6:**
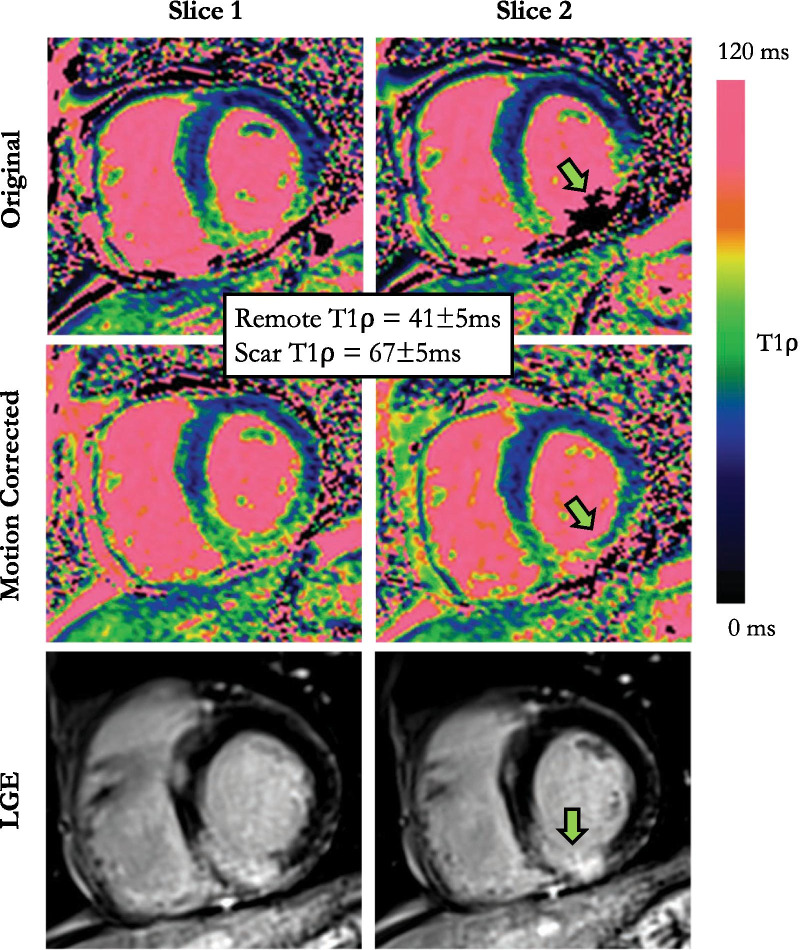
64-year-old male patient with basal and mid infero-septal and inferior transmural myocardial infarction on LGE. Non-corrected maps show large motion artefacts on the area of injury (green arrow). Motion-corrected T1ρ maps show sharper images with T1ρ elevation on the corresponding areas of injury on LGE (injury: 67 ± 5 ms vs. remote: 41 ± 5 ms)

The respiratory excursion of the heart, as assessed from the MPD across T1ρ-weighted images without motion correction, was much higher in patients than in healthy subjects (5.1 ± 2.7 mm vs. 0.5 ± 0.4 mm, P < 0.01, Fig. 4A). The addition of motion correction efficiently corrected for this motion, with higher DSC scores (87.7 ± 5.3% with motion correction vs. 82.2 ± 7.5% without, P < 0.01), and lower MPD (3.5 ± 1.9 mm vs. 5.1 ± 2.7 mm, P < 0.01). DSC and MPD scores obtained on a slice and T1ρ-weighting level are provided in Additional file 3. The inter-observer variability was found to be excellent for both DSC measurements [intraclass correlation coefficient: ICC = 0.88 (95% confidence interval 0.75–0.93), mean bias − 1.8% (95% limits of agreement − 8.7 to + 5.1%)] and MPD [ICC = 0.90 (95% confidence interval 0.75–0.95), mean bias − 0.4 mm (95% limits of agreement − 2.3 to + 1.5 mm)]. Consequently, T1ρ values increased after motion correction (mean T1ρ 48.8 ± 6.5 ms vs. 45.9 ± 6.3 ms, P = 0.02), whereas the precision of T1ρ values was similar (T1ρ SD 4.2 ± 1.2 ms vs. 4.2 ± 1.3 ms, P = 0.93). Additional file 4 plots the evolution of remote and injured T1ρ values as a function of iteration numbers and where the convergence of the proposed motion correction technique can be observed. Image quality of the T1ρ maps was significantly improved after motion correction (3.6 ± 0.6 vs. 2.1 ± 0.9, P < 0.01). It was graded as excellent for 19/30 (63%) of the motion corrected T1ρ maps, and 2/30 (7%) of the non-corrected T1ρ maps. No dataset was deemed non diagnostic (score 1) in the motion-corrected images whereas 9/30 (30%) of the non-corrected maps were graded as nondiagnostic. The benefit of correcting for respiratory motion can be appreciated in Fig. 5. Representative examples of non-corrected and motion-corrected myocardial T1ρ maps are shown in Fig. 6.

### Significance of T1ρ abnormalities in patients

**Fig. 7:**
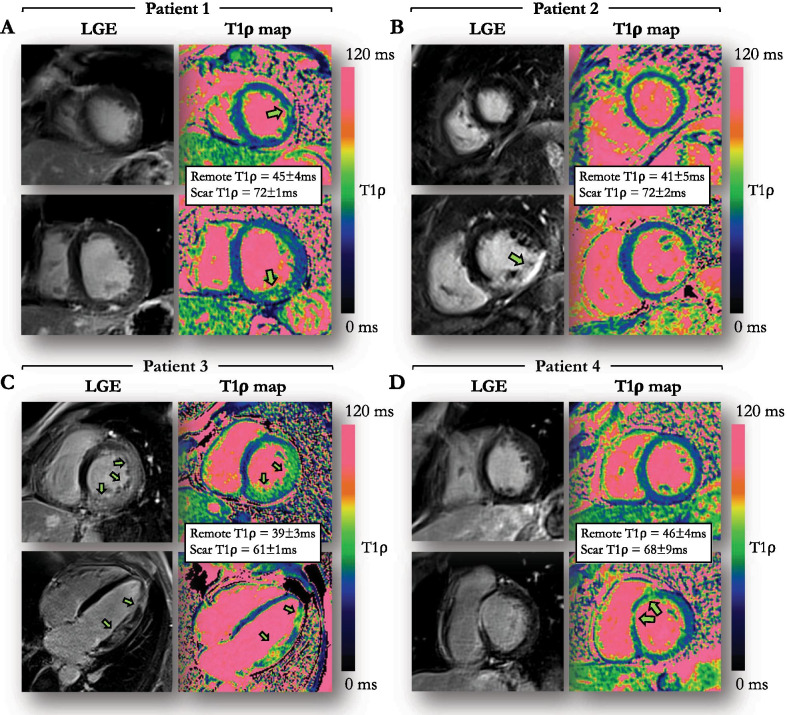
Examples of four patients with evidence of myocardial injury on LGE and motion-corrected T1ρ mapping. The regions of injury are indicated by arrowheads (**A**) 59-year-old male patient with sub-epicardial LGE in the infero-basal segment and intramural LGE on the latero-apical segment. **B** 53-year-old male patient with ischemic cardiomyopathy and transmural LGE in the inferior and infero-septal mid segments. **C** 51-year-old male patient with acute myocarditis and extensive patchy intramural and subepicardial LGE in the left ventricular free wall. **D** 35-year-old male patient with myocarditis and intramural LGE in the antero-septo-basal segment

Regionally increased T1ρ on motion-corrected myocardial T1ρ mapping was found in 15 (50%) patients. T1ρ could be compared to LGE findings in 23/30 patients only, because the position of T1ρ slices (which was performed in a standardized manner and without knowledge of the LGE positioning) did not cover LGE-containing slices in seven patients. In these 23 patients, including 15/23 LGE positive, the sensitivity and specificity of T1ρ to identify LGE were 93.3% [95% confidence interval 73.6–100.0%] and 88.9% [95% confidence interval 68.2–98.9%], respectively. Examples illustrating excellent agreement between T1ρ value elevation and LGE are shown in Fig. 7. Myocardial injuries were not detected on T1ρ maps despite positive LGE in one patient. These consisted in small focal intramural fibrosis patches on anterior and posterior right ventricle insertions in a patient with dilated cardiomyopathy and pulmonary hypertension. Conversely, one patient showed positive T1ρ findings despite negative LGE. This consisted of a large anteroapical area of increased T1ρ in a patient with acute takotsubo cardiomyopathy. Regarding T1ρ measurements, T1ρ values in injured (LGE positive) areas were significantly higher than in the remote myocardium (68.4 ± 7.9 ms vs. 48.8 ± 6.5 ms, P < 0.01) (Fig. 4B), while T1ρ values in the remote myocardium were similar to those measured in healthy subjects (P = 0.65). Regarding edema, T1ρ imaging could be assessed in all four patients with T2-positive injuries and was positive in all (3 acute myocarditis and 1 Takotsubo cardiomyopathy). Regarding cine imaging, wall motion was found to be abnormal in 11/15 T1ρ positive lesions and was preserved in the remaining 4/15.

## Discussion

The main findings of this study are that

1. although the residual motion during breath-held myocardial T1ρ mapping acquisitions can be considered negligible in healthy subjects, it is much larger in patients, and significantly impacts the quality of T1ρ maps,
2. model-based non-rigid motion correction can be effectively applied to address this issue and improve the quality of myocardial T1ρ maps,
3. motion-corrected T1ρ mapping may allow for the detection of acute and chronic myocardial injuries of various origins in patients, without the need for exogenous contrast agents.

In practice, robust non-rigid motion correction of pre-contrast myocardial T1ρ maps has been poorly addressed until now. The robustness is particularly needed when registering images with large contrast differences such as the first and last spin lock points (i.e., TSL = 0 ms and TSL = 50 ms) where conventional deformable registration algorithms could fail. Previous studies, also making use of breath-holding, made the assumption that residual motion artefacts caused by poor breath-holding are negligible for short breath-holds [27, 28]. In reality, we have observed that most patients fail to properly hold their breath, even for short breath-hold durations of less than 13 s. In patients, we observed respiration displacement of the heart of 5.1 ± 2.7 mm, confirming the need for using advanced motion correction techniques.

In this pilot study, we demonstrate the feasibility of an inline non-rigid motion corrected myocardial T1ρ mapping framework to robustly quantify myocardial injury without the need for exogenous contrast agent. We highlight the importance of simultaneously performing joint non-rigid registration and T1ρ model fitting to deal with multi-contrast T1ρ-weighted images and to address the need of providing broader adoption of 2D myocardial T1ρ mapping in clinical practice.

The performance of the proposed framework was first evaluated on an ex vivo human heart with a motion-controlled experimental setup and further assessed in vivo in eight healthy subjects and 30 patients with a broad spectrum of ischemic and non-ischemic injuries. An essential part of the technique is the complete automation of the reconstruction with fast inline reconstruction (~ 30 s). This is crucial for direct image analysis, clinical acceptance and future widespread clinical use.

The proposed strategy pools the pair-wise non-rigid motion correction and T1ρ fitting in a unified optimization framework. As compared to non-corrected images, the non-rigid model-based motion correction significantly improves the T1ρ map quality. By exploiting the signal model, the method becomes robust to contrast change, as opposed to a standard intensity-based deformable registration [9, 10] and pair-wise registration using mutual information as similarity criterion (Additional files 5, 6). The proposed technique, while optimized for myocardial T1ρ mapping, is not limited to this single-shot sequence and may easily be extended to other cardiac mapping sequences such as T1 and T2 mapping.

Other model-based registration approaches could also be employed for myocardial T1ρ mapping. For example, the technique proposed by Hue et al. [14], which combines variational energy minimization with pair-wise registration, was shown to be efficient to reconstruct motion-free inversion recovery-based myocardial T1 maps. Although, similarities can be found with our approach, the image contrast, dynamic range and noise level given by inversion recovery-based sequences are substantially different than the ones observed on T1ρ-weighted images. A comparison of the different techniques could be explored in future work after careful consideration of the above-mentioned effects.

Moreover, while a simple mono-exponential signal model was considered in this pilot study, more advanced Bloch equation or extended phase graph simulations could be integrated into the reconstruction framework. By taking into consideration more information about the sequence, such as magnetization transfer, off-resonance, B1 inhomogeneities, slice profile and other complicating factors, dictionary-based simulations would provide more accurate and precise myocardial T1ρ maps.

We have observed in patients that respiratory motion of the heart, when not corrected, can considerably deteriorate the quality of the reconstructed T1ρ maps, making LV segmental analysis highly unreliable. In patients, DSC scores were higher, MPD were lower, and image quality was improved after motion correction.

Our patient study was not designed to assess the accuracy of myocardial T1ρ mapping in detecting myocardial injuries as compared to conventional LGE imaging. However, similar to previous studies [5, 11, 29], we observed a significant T1ρ elevation in patients with myocardial injuries of various origin (47% increase in value). These results are consistent with findings from Stoffers et al. [11] where the authors found a T1ρ difference between infarcted and remote myocardial tissue in a swine model of 68.1 ms. Likewise, an increase of 44.5 ms in T1ρ values was observed by Witschey et al. in swine undergoing post-surgical induction of myocardial infarction [5]. In patients with chronic myocardial infarction, a prior study showed higher T1ρ values in the infarct region as compared to remote areas (79 ± 11 ms vs. 54 ± 6 ms) [29]. This is consistent with our findings, T1ρ being able to identify LGE areas with 93.3% sensitivity and 88.9% specificity in our series. Interestingly, the two cases showing a discrepancy between T1ρ and LGE imaging may be explained. The false negative case was likely due to the small lesion size and potential partial volume averaging. The false positive case corresponded to a Takotsubo cardiomyopathy with abnormal T2 and T1ρ values despite negative LGE, potentially indicating a sensitivity of T1ρ to non-necrotizing myocardial injuries. These findings confirm the high potential of myocardial T1ρ mapping as a gadolinium-free CMR technique for the detection and characterization of structural heart diseases. The contrast-free nature of the method may promote novel CMR applications for the screening of asymptomatic subjects.

### Study limitations

The study has several limitations. Firstly, for practical reasons, this study only included a limited number of patients and, in these, T1ρ mapping was only performed on 3 discrete slices, as opposed to a whole LV coverage on LGE imaging. Because T1ρ mapping was performed pre-contrast without any prior knowledge about presence, extent, and location of scar, T1ρ data was not available in a significant number of patients with LGE lesions. In addition, only a small number of acute injuries exhibiting high T2 values were available for comparison. Larger clinical studies are required to further validate the proposed motion-corrected T1ρ mapping technique and to assess its sensitivity, specificity and diagnostic value in patients with chronic and acute myocardial injuries. In particular, infarct location, size, extent, and transmurality are features that would ultimately need to be compared between T1ρ mapping and standard LGE imaging. Comparisons with conventional T1 and T2 mapping techniques will also have to be consistently performed to further elucidate the added value of T1ρ mapping and to better understand whether this may provide us with complementary quantitative information on myocardial tissue composition. Another limitation lies in the lack of a reference standard, such as histology. In the present study, an ex vivo human heart was used to optimize motion correction parameters, but the infarct present in this organ could not be considered as a reference for T1ρ in scar, due to changes induced by the fixation and preservation protocol. Thus, the performance of our technique in the assessment of myocardial injuries was limited to the comparison to conventional post-contrast LGE imaging in patients.

A last set of limitations is related to the proposed model-based non-rigid motion correction framework, which cannot account and correct for through-plane motion but only residual in-plane respiratory motion. We acknowledge that residual through-plane motion may also affect the quality of the reconstructed T1ρ map. Solutions to this problem may include real-time slice tracking using diaphragmatic navigators. However, as the signal intensity at the lung-liver interface depends on the variable spin lock times, straightforward solutions may not easily be found. An alternative and most obvious solution includes 3D T1ρ mapping [30–32]. The larger 3D coverage may also help visualizing more and smaller injuries, while the use of advanced self-gated navigation techniques [33] will facilitate the application of the technique in patients having difficulties holding their breath. Further improvements in image quality could also be achieved by integrating the estimated non-rigid respiratory motion fields directly in the image reconstruction process [34, 35].

## Conclusions

The proposed myocardial T1ρ mapping framework with model-based non-rigid motion correction enables a quantitative characterization of myocardial injuries with relatively low sensitivity to respiratory motion. This technique may be a robust and contrast-free adjunct to LGE for gaining new, additional, and quantitative insight into acute and chronic myocardial structural disorders.

## Supplementary Information

**Additional file 1.** Comparisons of non-adiabatic and adiabatic T1ρ-prepared single-shot images in a healthy subject.

**Additional file 2.** Examples of T1ρ-weighted images and corresponding T1ρ maps before and after motion correction in two patients with high (top row) and low (bottom row) reduction of maximum perpendicular distance.

**Additional file 3.** Dice scores (DSC) and maximum perpendicular distance (MPD) obtained in patients before and after model-based non-rigid motion correction on a T1ρ-weighted image level (A, B) and on a short-axis slice level (C, D).

**Additional file 4.** Convergence of the proposed model-based non-rigid motion correction algorithm for myocardial T1ρ mapping.

**Additional file 5.** Comparison of the proposed model-based non-rigid registration with standard deformable registration in a patient.

**Additional file 6.** Visual comparisons of the proposed model-based non-rigid registration with a pair-wise registration using mutual information as similarity criterion for myocardial T1 rho mapping in two patients.

## Data Availability

The acquired datasets and the reconstruction code used in this study are available from the corresponding author on reasonable request.

## Abbreviations

bSSFP: Balanced steady state free precession
CMR: Cardiovascular magnetic resonance
DSC: Dice similarity coefficient
ECG: Electrocardiogram
FSL: Spin lock frequency
LGE: Late gadolinium enhancement
LV: Left ventricle/left ventricular
LVEF: Left ventricular ejection fraction
MPD: Maximum perpendicular distance
RF: Radiofrequency
SL: Spin lock
TSL: Spin lock time
