# Supplementary figures and images for: Endogenous assessment of myocardial injury with single-shot model-based non-rigid motion-corrected T1 rho mapping
Source: J Cardiovasc Magn Reson. 2021 Oct 21;23:119. doi: 10.1186/s12968-021-00781-w (PMC8529795; doi:10.1186/s12968-021-00781-w)

Non-adiabatic T1 $\rho$  pulse

Adiabatic T1 $\rho$  pulse

— Subject 1 —

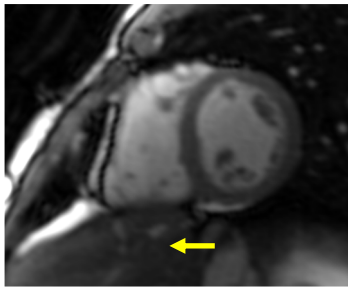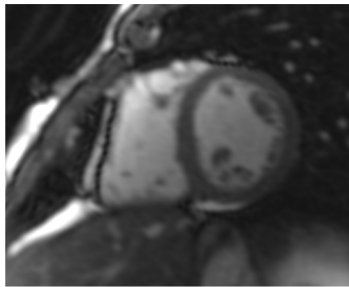

— Subject 2 —

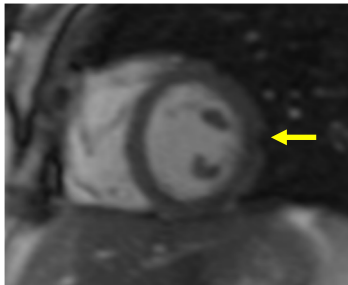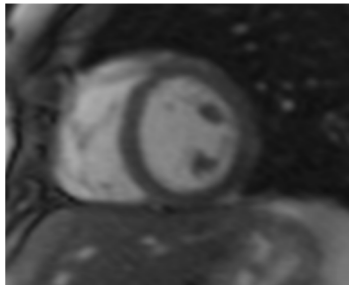

Supplement: Supplementary file 1 — Additional file 1. Comparisons of non-adiabatic and adiabatic T1ρ-prepared single-shot images in a healthy subject. [file 12968_2021_781_MOESM1_ESM.pdf]

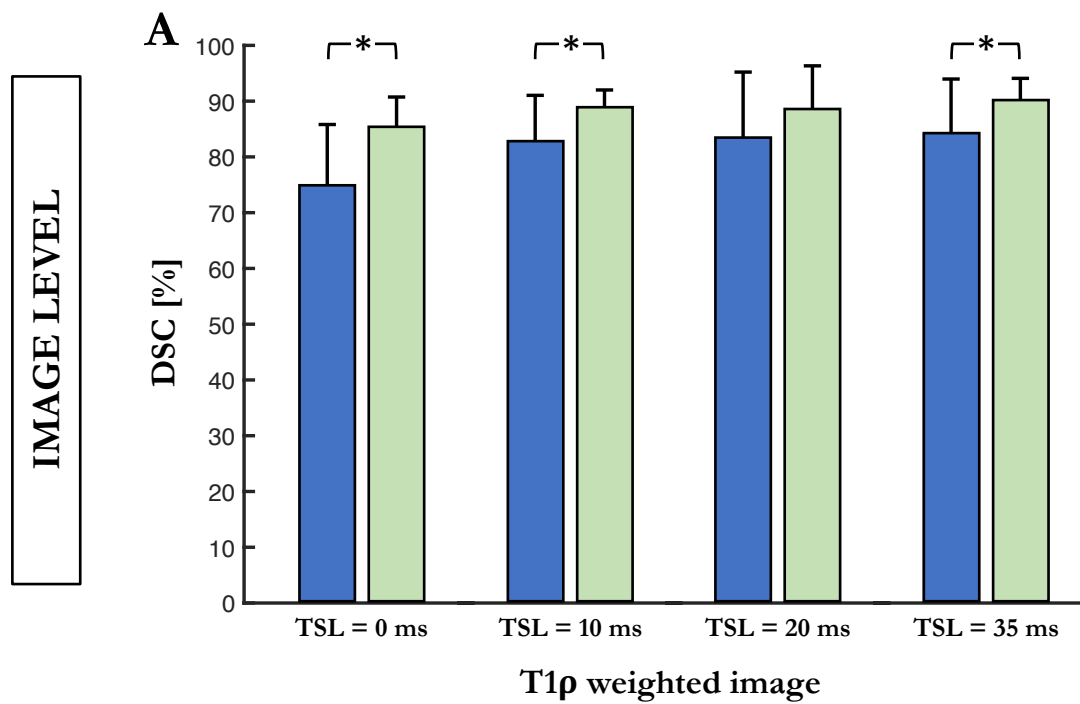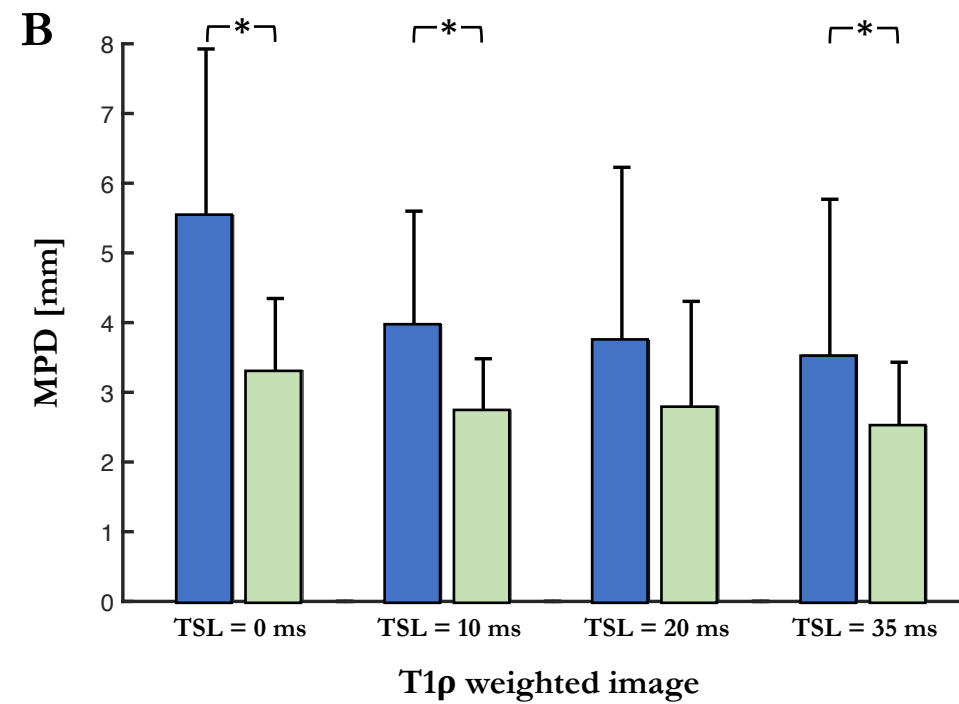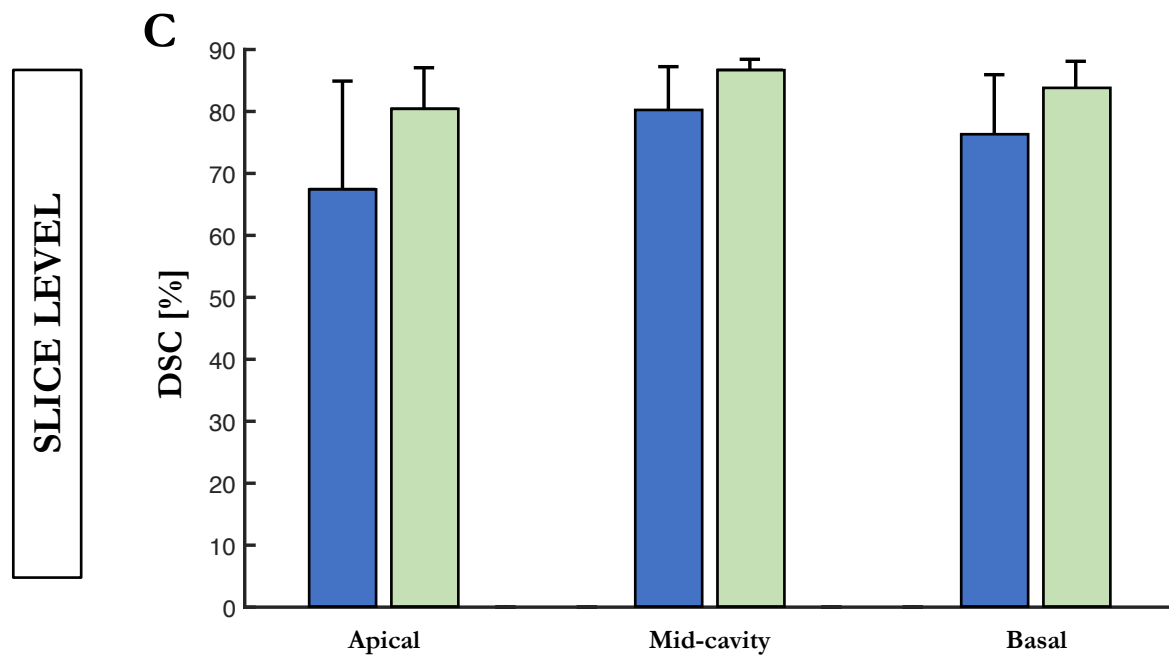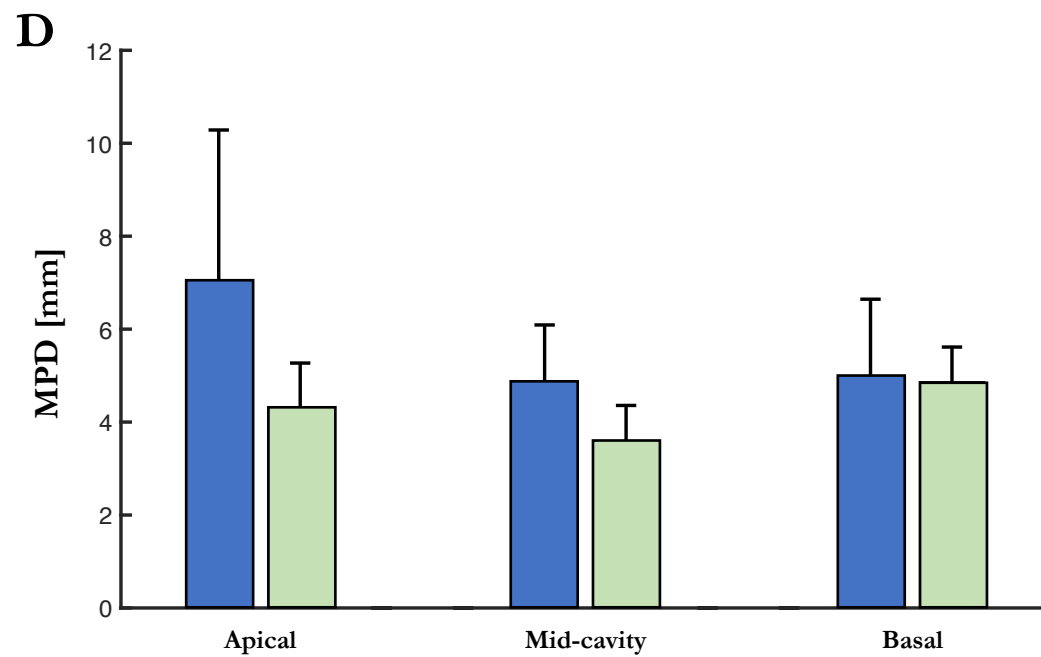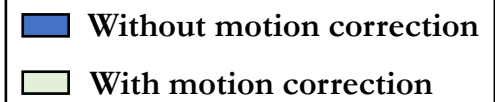

Supplement: Supplementary file 3 — Additional file 3. Dice scores (DSC) and maximum perpendicular distance (MPD) obtained in patients before and after model-based non-rigid motion correction on a T1ρ-weighted image level (A, B) and on a short-axis slice level (C, D). [file 12968_2021_781_MOESM3_ESM.pdf]

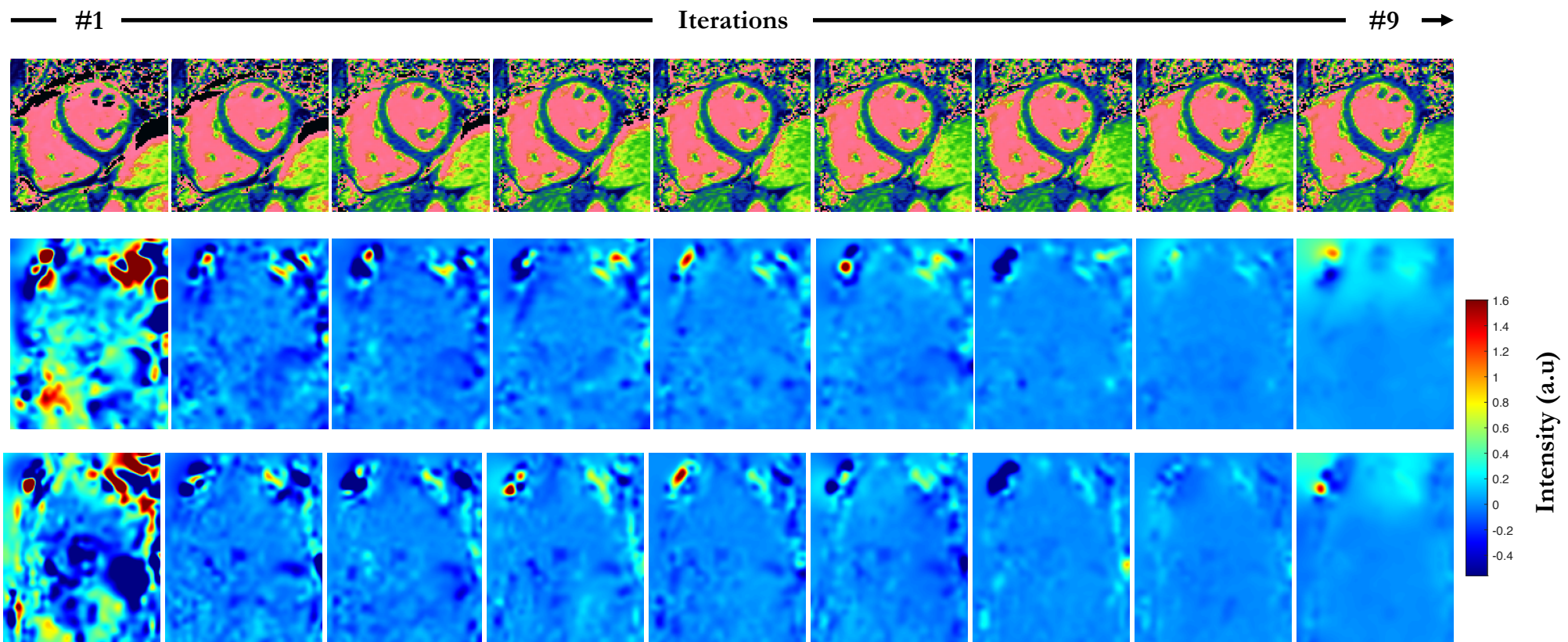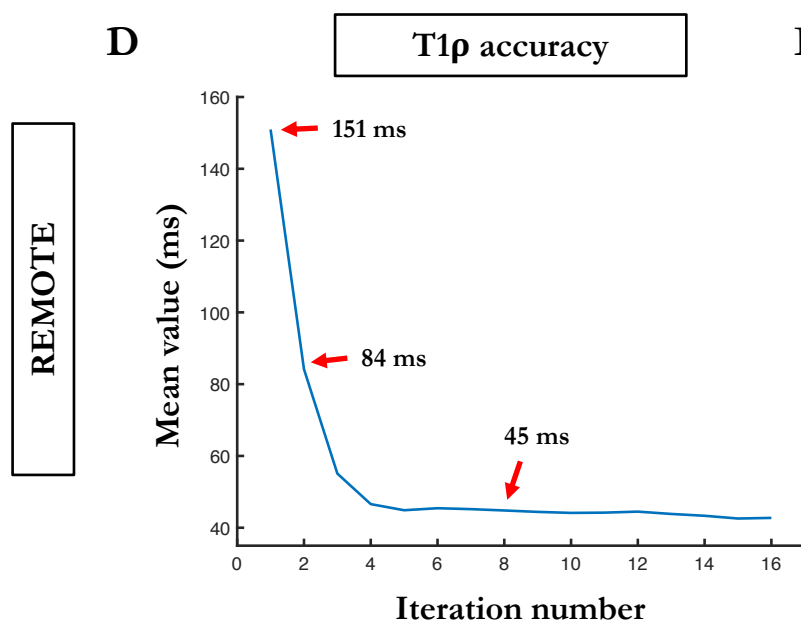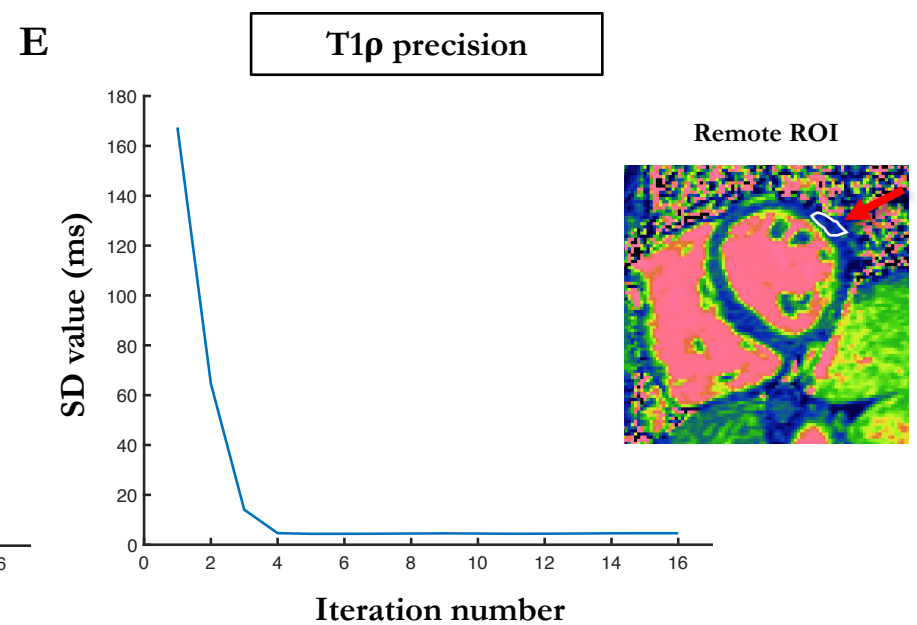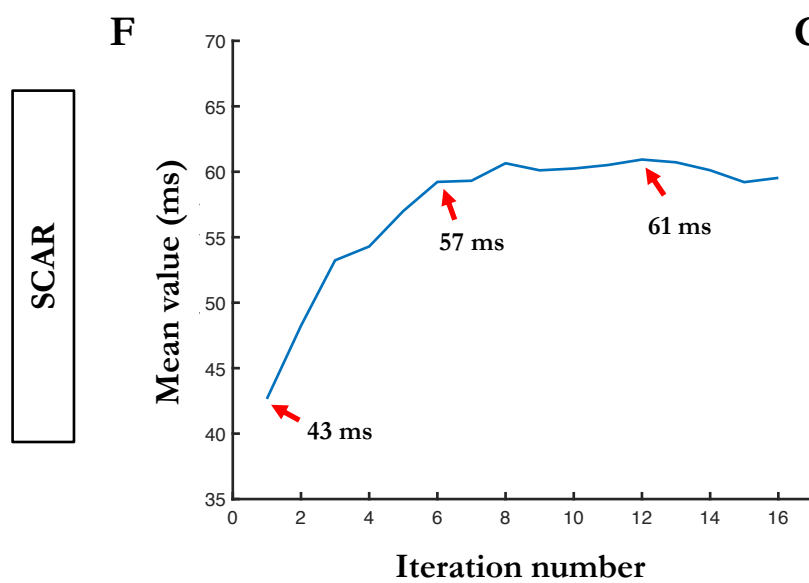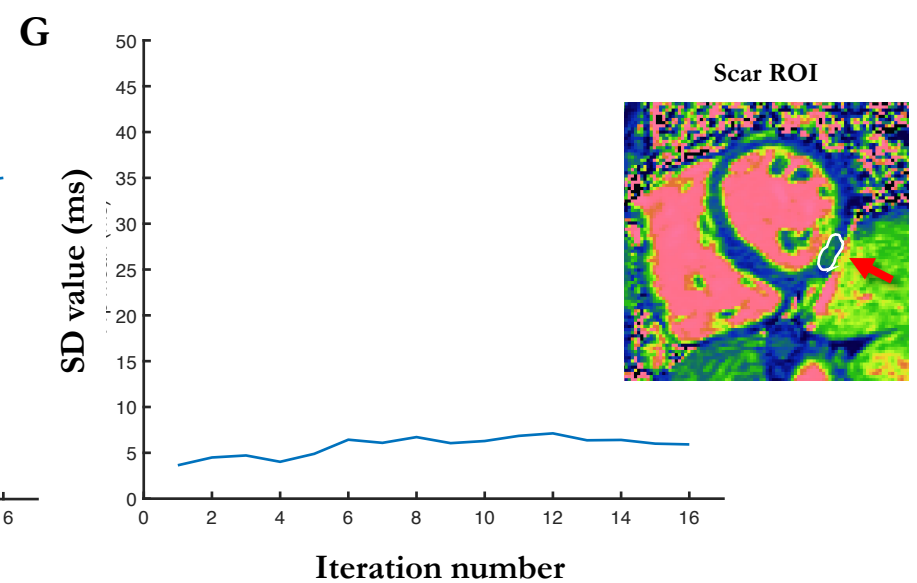

Supplement: Supplementary file 4 — Additional file 4. Convergence of the proposed model-based non-rigid motion correction algorithm for myocardial T1ρ mapping. [file 12968_2021_781_MOESM4_ESM.pdf]

TSL = 0ms

TSL = 10ms

TSL = 20ms

TSL = 35ms

TSL = 50ms

T1 $\rho$  map

120 ms

T1 $\rho$ 

0 ms

Uncorrected

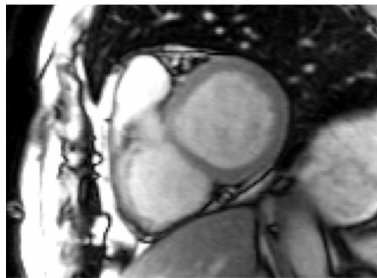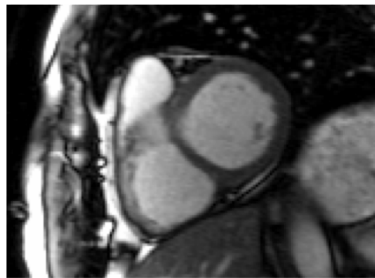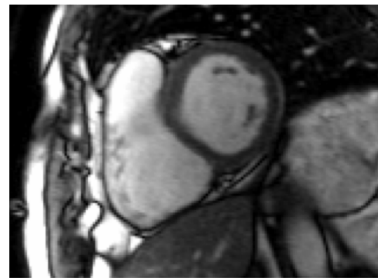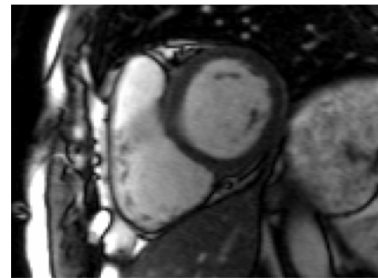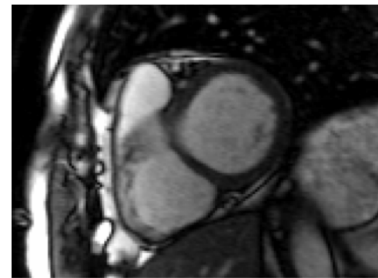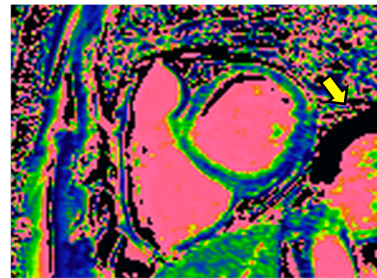

Deformable

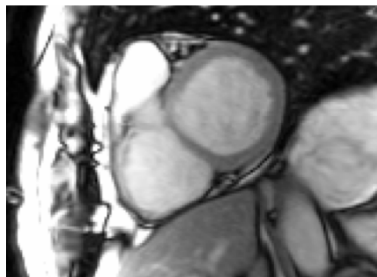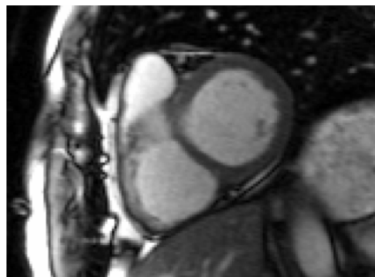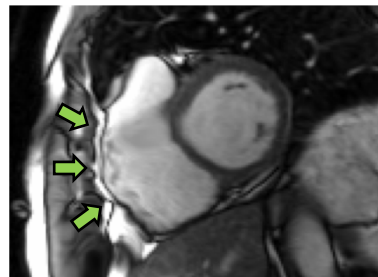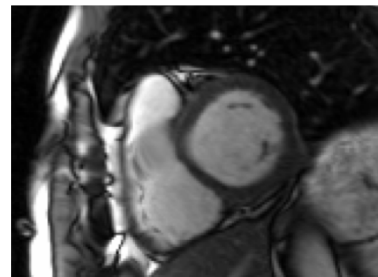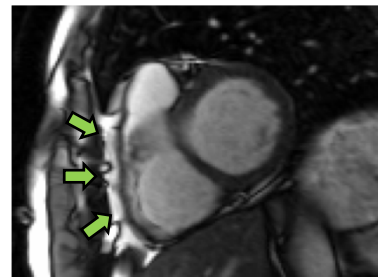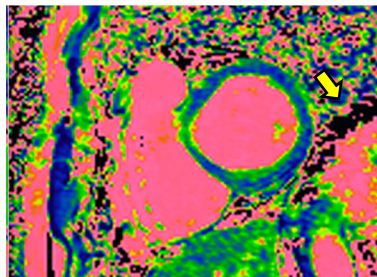

Model-based

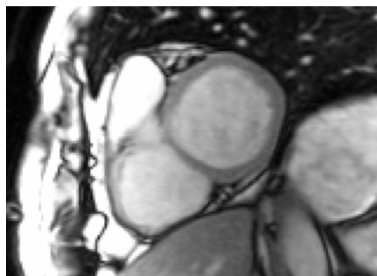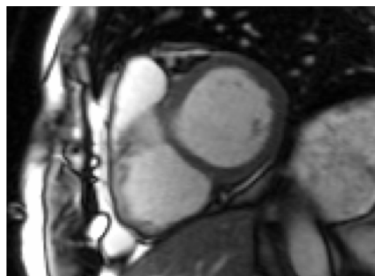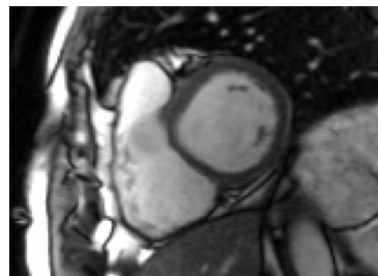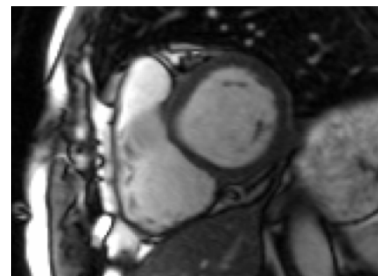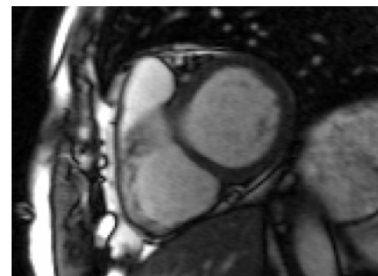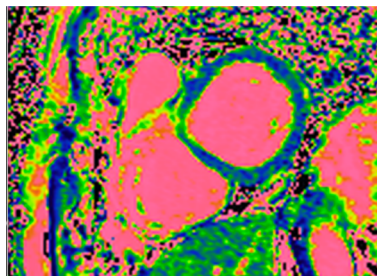

Supplement: Supplementary file 5 — Additional file 5. Comparison of the proposed model-based non-rigid registration with standard deformable registration in a patient. [file 12968_2021_781_MOESM5_ESM.pdf]

Patient 5

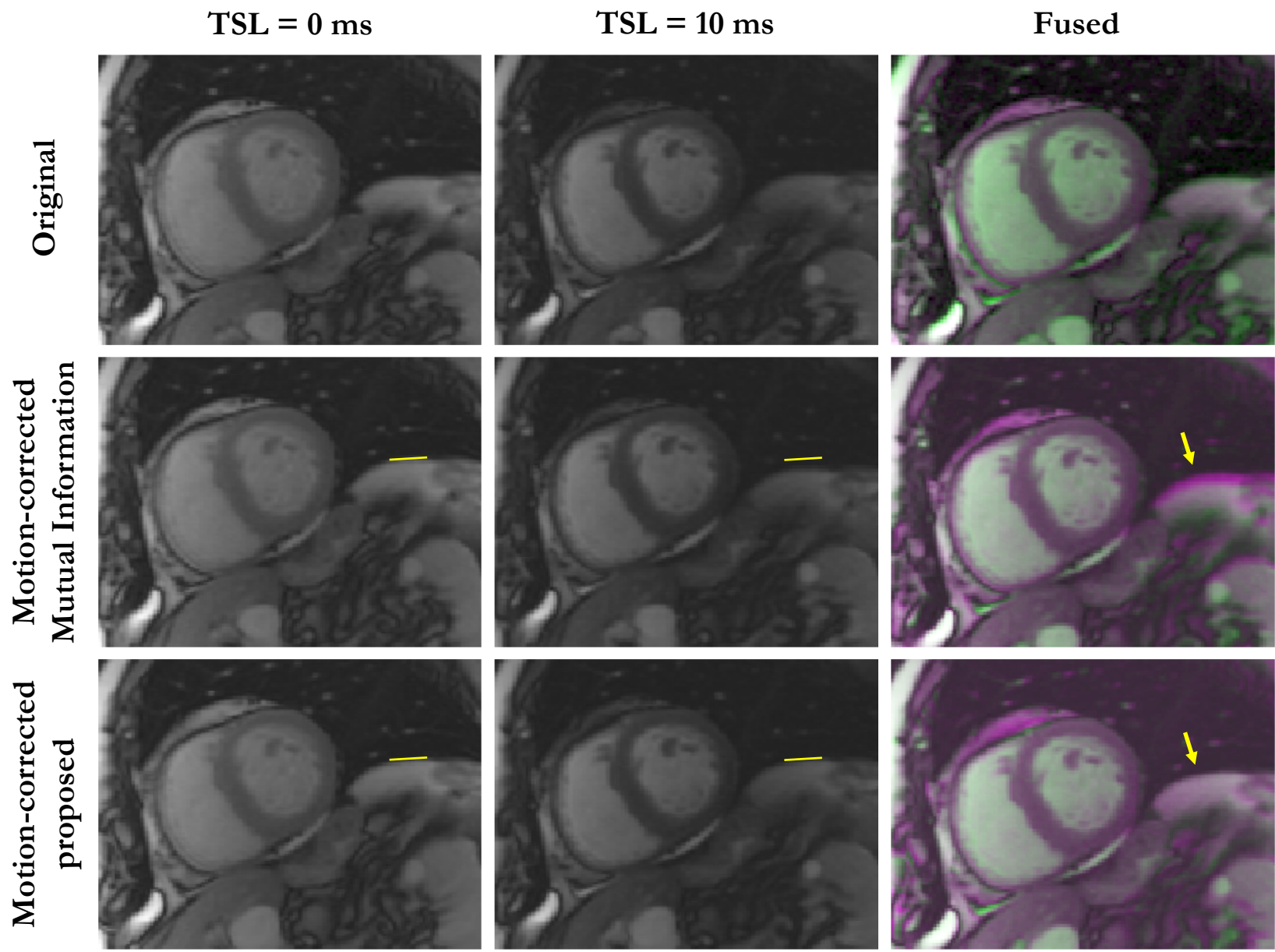

Patient 6

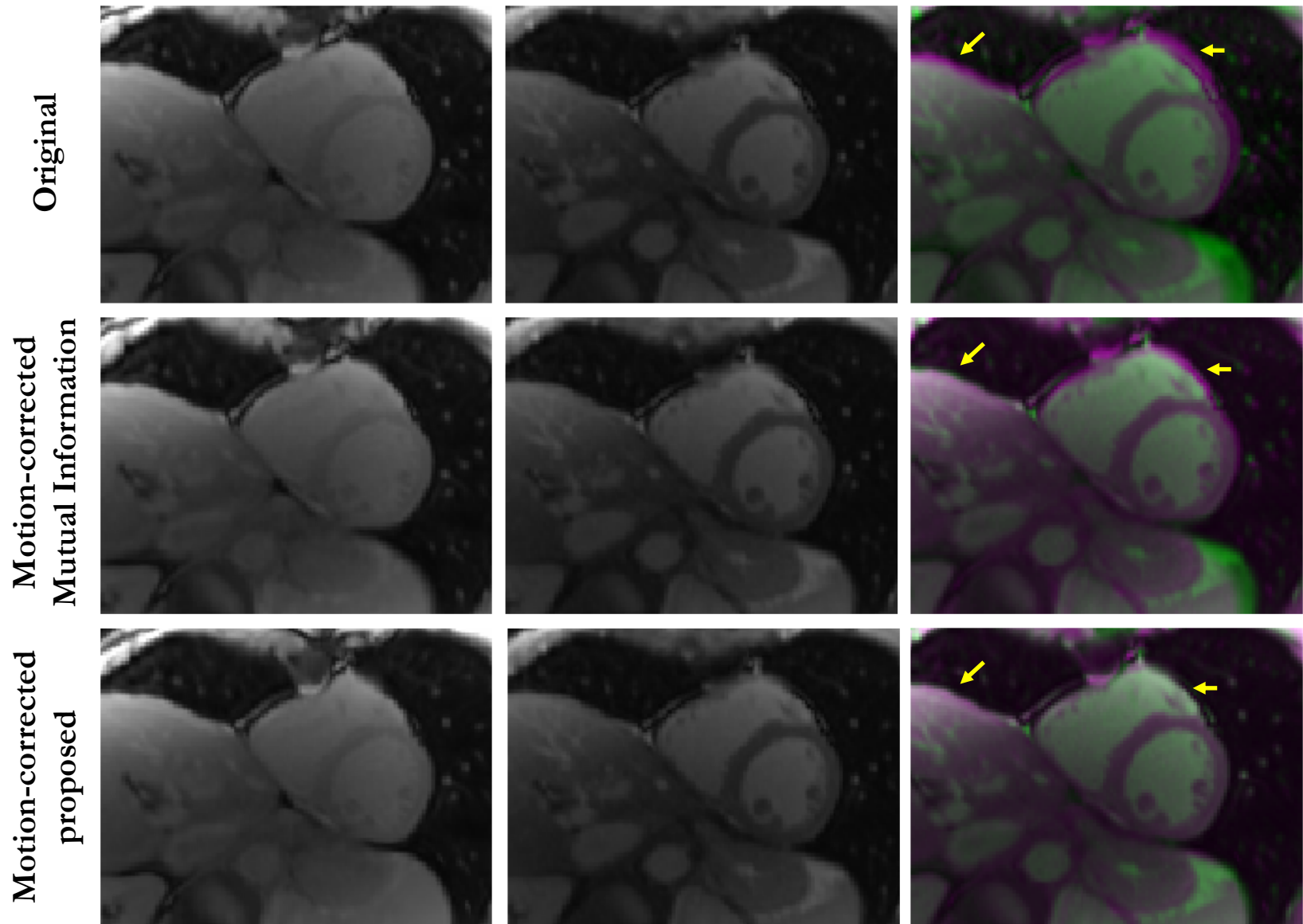

Supplement: Supplementary file 6 — Additional file 6. Visual comparisons of the proposed model-based non-rigid registration with a pair-wise registration using mutual information as similarity criterion for myocardial T1 rho mapping in two patients. [file 12968_2021_781_MOESM6_ESM.pdf]
