# Supplementary material for: Endogenous assessment of myocardial injury with single-shot model-based non-rigid motion-corrected T1 rho mapping
Source: J Cardiovasc Magn Reson. 2021 Oct 21;23:119. doi: 10.1186/s12968-021-00781-w (PMC8529795; doi:10.1186/s12968-021-00781-w)
Supplement: Supplementary file 2 — Additional file 2. Examples of T1ρ-weighted images and corresponding T1ρ maps before and after motion correction in two patients with high (top row) and low (bottom row) reduction of maximum perpendicular distance. [file 12968_2021_781_MOESM2_ESM.pdf]

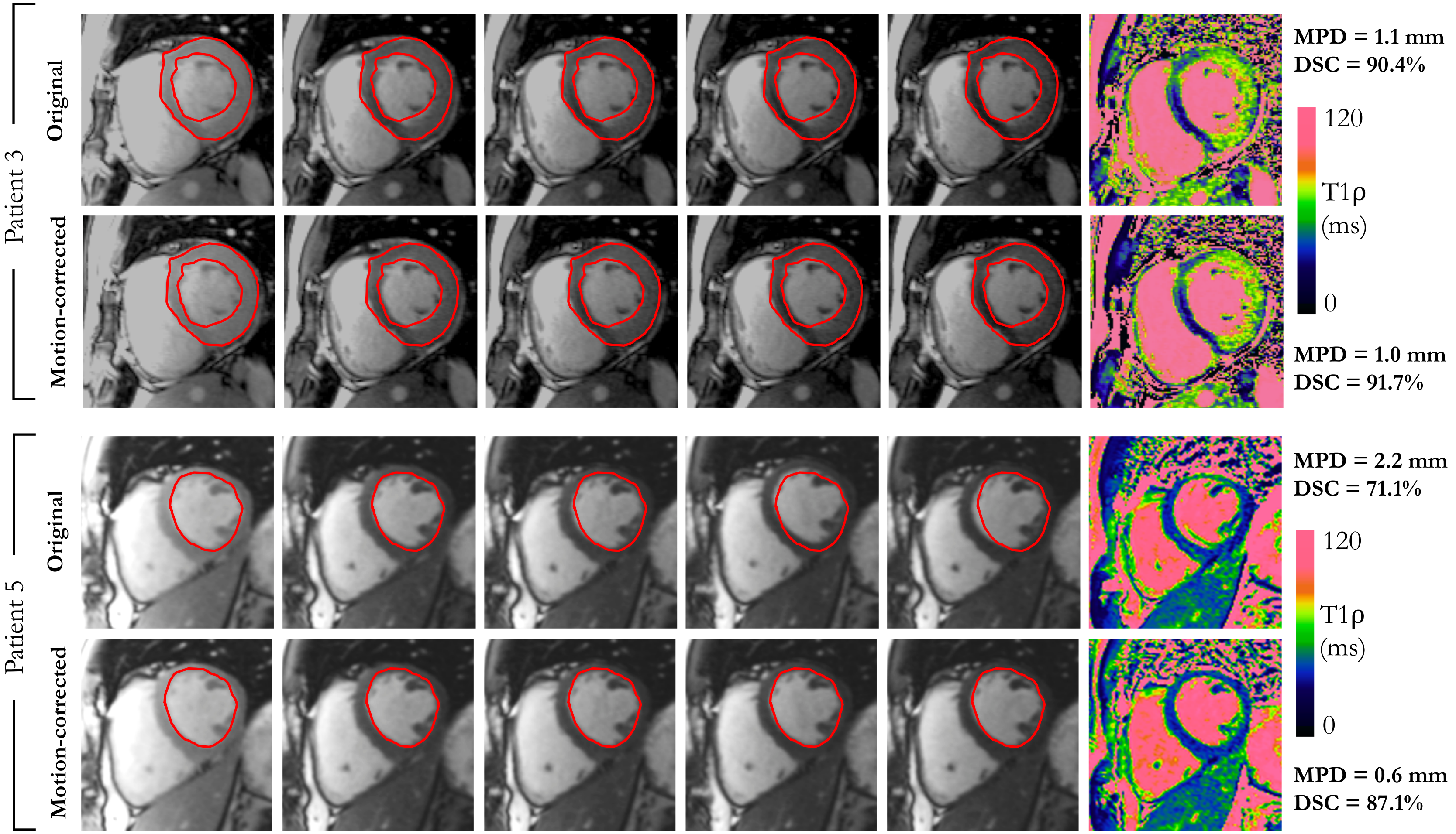
Patient 3
Original
Motion-corrected
MPD = 1.1 mm
DSC = 90.4%
120
T1ρ
(ms)
0
MPD = 1.0 mm
DSC = 91.7%
Patient 5
Original
Motion-corrected
MPD = 2.2 mm
DSC = 71.1%
120
T1ρ
(ms)
0
MPD = 0.6 mm
DSC = 87.1%
